# Teacher Support and Mental Well-Being in Chinese Adolescents: The Mediating Role of Negative Emotions and Resilience

**DOI:** 10.3389/fpsyg.2019.03081

**Published:** 2020-01-22

**Authors:** Junqiao Guo, Ling Liu, Bihua Zhao, Daoyang Wang

**Affiliations:** ^1^School of Educational Science, Anhui Normal University, Wuhu, China; ^2^Collaborative Innovation Center of Assessment Toward Basic Education Quality, Beijing Normal University, Beijing, China

**Keywords:** teacher support, mental well-being, negative emotions, resilience, adolescents

## Abstract

Teacher support has been shown to enhance adolescent mental health. However, the effects of negative emotions and resilience in the relationship between teacher support and mental well-being in adolescents are still unknown. This study investigated (a) the mediating role of negative emotions in the relationship between teacher support and mental well-being, (b) the mediating role of resilience in the association between teacher support and mental well-being, (c) the serial mediating role of negative emotions and then resilience in the relationship between teacher support and mental well-being, and (d) the parallel mediating role of the five dimensions of resilience and the three factors of negative emotions in the relationship between teacher support and mental well-being. Participants were 1228 Chinese adolescents (age, *M* = 15.43 years; 53.09% male). Participants filled out questionnaires regarding teacher support, negative emotions, resilience, and mental well-being. After controlling for age and gender, we found that teacher support, negative emotions, and resilience were significantly linked with mental well-being. Moreover, negative emotions and resilience were found to mediate the relationship between teacher support and adolescent mental well-being, accounting for 5.45 and 30.00% of the total effect, respectively. We also found that teacher support enhances mental well-being by decreasing negative emotions and then increasing resilience. This serial mediating effect accounted for 8.48% of the total effect. Finally, the mediating effect of resilience between teacher support and mental well-being was significantly greater than the mediating effects of the other two indirect effects (negative emotions in the relationship between teacher support and mental well-being, negative emotions and then resilience in the relationship between teacher support and mental well-being). And the parallel mediation model showed that teacher support can promote adolescent mental well-being by increasing goal planning, affect control, and help-seeking behavior, and decreasing depression. These findings suggest a process through which negative emotions can decrease resilience and identify the mediating effects of negative emotions (including the three dimensions of negative emotions) and resilience (including the five factors of resilience) in the relationship between teacher support and adolescent mental well-being.

## Introduction

Mental well-being is a dynamic state that allows individuals to be happy and satisfied with life, find purpose in their lives, realize their given potential, form and maintain relationships with others, and feel in control of their own lives (Ryff and Keyes, 1995). It is usually used interchangeably with positive mental health, which is a complex theoretical construct, covering both hedonic (happiness, life satisfaction) and eudaimonic (self-realization, psychological functioning) well-being (Ryan and Deci, 2001; Tennant et al., 2007; Lang and Bachinger, 2017). Clarke et al. (2011) pointed that the promotion of mental well-being has become a national priority for children and adolescents in United Kingdom. Given its importance for all aspects of life, mental well-being has received increasing attention (Dong et al., 2016; Ng et al., 2017). Research has identified several variables that influence mental well-being, including resilience, depression, anxiety, and social support (Liu et al., 2009; Strange et al., 2015; Khawaja et al., 2017). However, more study is necessary to deepen our understanding of the mechanisms underlying the relationship between social support from teachers and mental well-being (Guo et al., 2018). Furthermore, mental well-being is important for the healthy development of adolescents (Singh et al., 2015). Therefore, the current study investigated the association between teacher support and mental well-being of adolescents, as well as the influence of negative emotions and resilience in this relationship.

### The Relationship Between Teacher Support and Mental Well-Being

According to some researchers, mental well-being is an aspect of mental health. For instance, Weich et al. (2011) wrote, “take mental health to mean the full spectrum of mental health states; mental illness refers to pathological disease states and mental well-being covers the positive end of the spectrum (p. 23).” Thus, the main effect model of social support, which describes the relationship between social support and mental health, should apply to mental well-being. This model indicates that social support can help people stay healthy and feeling good in all kinds of circumstances, and the increase of social support will facilitate the improvement of individual health status, no matter what the current level of support is (Cohen and Wills, 1985; Gong, 1994). One study reported that the more social support the caregivers of schizophrenia patients perceive, the better their mental health (Lee et al., 2006).

The ecological model of adolescent development specifies that adolescents’ health and well-being are affected by some mutually interacting environmental settings, especially the microsystem level, including adolescents’ direct interactions with teachers, parents, friends, and others in their immediate environment (Bronfenbrenner, 1986; Ferguson et al., 2011). In the context of schools, teachers can provide adolescents with different dimensions of support, such as emotional support, academic support, and competence support (Ahmed et al., 2010; Vervoort et al., 2014; Ansong et al., 2017; Liu et al., 2018), which are beneficial for the development of adolescents. Hence, more studies should investigate the effect of social support offered by teachers. According to the main effect model of social support (Cohen and Wills, 1985; Gong, 1994) and the ecological model of adolescent development (Bronfenbrenner, 1986), teacher support may be a protective factor for adolescent mental well-being. For instance, some studies found that support from teachers has a positive influence on adolescent psychological well-being, mental health, happiness, and satisfaction (Lau et al., 2010; Wang et al., 2014; Alivernini et al., 2019). Although many studies have demonstrated the association between teacher support and mental illness, such as depression (Pössel et al., 2018) and anxiety (Zhao and Li, 2017), only a few studies have explored the positive association between teacher support perceived by middle school students and positive mental health (Guo et al., 2018). More importantly, the mechanism underlying these protective resources and positive outcomes has yet to be identified. Thus, it is necessary to more fully investigate the relationship between teacher support and adolescent mental well-being, and to identify the contributions of different resources such as resilience (protective resource) and negative emotions (destructive resource) to this association.

### The Mediating Role of Resilience in the Relationship Between Teacher Support and Mental Well-Being

Resilience is a very complex construct (Southwick et al., 2014) and it can be viewed as a trait (Hu et al., 2015; Calegaro et al., 2019), a process (Panter-Brick and Leckman, 2013; Stainton et al., 2018), or an outcome (Masten, 2014). In this study, resilience is defined both as an inner psychological potential and as a dynamic process of coping with disruptive, stressful, or challenging life events in a healthy way at minimal physical and psychological cost (Richardson et al., 1990; Luthar, 1991; Epstein and Krasner, 2013; Stainton et al., 2018). The positive factors of resilience in adolescents are assets and resources (Fergus and Zimmerman, 2005). Assets refer to the internal protective resources that reside within the individual, such as self-efficacy, competence, and coping skills. Resources are the positive factors that are external to the individual, such as parental support or adult mentoring (Fergus and Zimmerman, 2005; Stainton et al., 2018).

According to the framework of resilience in action, when the external resources (including close relationships, high expectations, and positive participation from school, family, society, and peer groups) meet the psychological needs of adolescents, including safety, love, and belonging (Li and Zhang, 2006), they can be transformed into internal resources, such as self-efficacy, self-consciousness, and self-awareness, that aid adolescent development and personal growth. At the same time, resilience is also increased in the process of developing internal resources. This logic is similar with self-determination theory (Ryan and Deci, 2000), which points that individuals’ mental health and well-being are enhanced when their innate psychological needs including competence, autonomy, and relatedness are satisfied. Researchers found that adolescents who perceived respect and support from their parents or teachers have the highest levels of resilience (Gómez-Ortiz et al., 2015; Liebenberg et al., 2016). According to the two models and previous studies, teacher support can be a kind of external resource, which is extremely important to develop resilience in adolescents, further improving their mental well-being.

Research on teacher support has identified resilience as a potential mediator in the association between teacher support and adolescent mental well-being. Some studies have consistently found that good teacher–student relationships and communication can improve resilience (Morrison and Allen, 2007; Rosenberg et al., 2018). Resilience has also been identified as a protective factor against mental health issues, with one study reporting a correlation between resilience and mental health (Wilson and Saklofske, 2017; Wu et al., 2018). These findings suggest that resilience might account for the positive effect of teacher support on adolescent mental well-being.

### The Mediating Role of Negative Emotions in the Relationship Between Teacher Support and Mental Well-Being

Negative emotions are usually defined as negative emotional states, such as unpleasant or unhappy emotions that are evoked in individuals to express negative affect toward an event or person, and these usually include depression, anxiety, loneliness, anger, and stress (Cohen and Wills, 1985; as cited in Chen et al., 2016). In the current study, we used the short-form version of the Depression Anxiety Stress Scale (DASS-21, Lovibond and Lovibond, 1995) to measure the negative emotions, including depression, anxiety, and stress. The DASS-21 is an excellent tool which was conceptualized as a correlated three-factor model, and the total score of DASS-21 represents a full spectrum of negative emotional states (Lovibond and Lovibond, 1995; Chin et al., 2019; as cited in Prabhu, 2016). The buffering model of social support postulates that social support can maintain good health by decreasing the negative influence of stress on the body and mind (Gong, 1994). In line with this model, one study reported that older adults with low community social capital, such as social cohesion and community social ties, were vulnerable to depression when they faced stress (An et al., 2018). These results indicate that a lack of social support (i.e., teacher support) may lead to poor mental health by increasing negative emotional states, such as anxiety and depression. In other words, adolescents with higher levels of teacher support are more likely to perceive kindness and caring from teachers, and experience less negative emotions. Students who believe that they do things well in relationships with teachers display good normative adjustment and social adjustment (Herrera-López et al., 2016), which make them experience less negative emotions and feel better in the classroom. Based on the literature, it can be suggested that teacher support, as an important capital for adolescents, may antecede negative emotions, further facilitating adolescent mental well-being. In other words, students who perceive themselves as receiving less support from teachers may feel that they are worthless and unlovable; indeed, adolescents tend to have deficits in the self-system that manifest as low satisfaction of autonomy need and poor academic performance (Zhang et al., 2018). During this time, they may be more vulnerable to negative emotions. Studies have also demonstrated that students who frequently receive critical feedback from teachers and other school staff members are more likely to show higher levels of anxiety, educational stress, and depressive symptoms (Nguyen et al., 2013). However, teacher support can significantly reduce adolescent anxiety and depression (Yu et al., 2016). Furthermore, Extremera and Rey (2016) found that negative emotions are negatively associated with life satisfaction, which can be taken as an indicator of well-being. Although a direct examination of the associations between teacher support, negative emotions, and adolescent mental well-being is lacking, the existing literature indicates that teacher support reduces negative emotions in adolescents, which helps further develop their mental well-being.

### The Relationship of Negative Emotions and Resilience

As negative emotions and resilience have negative and positive relationship with mental health, respectively, it is reasonable to assume that these two constructs are associated with each other. Indeed, it has been reported that stress, depression, and anxiety have negative relationships with resilience (Haddadi and Besharat, 2010; Skrove et al., 2013; Anyan and Hjemdal, 2016; Yen et al., 2019). However, different studies have different views on the relationship between negative emotions and resilience. For instance, some researchers found that higher resilience predicted lower levels of depression, anxiety, and stress (Hjemdal et al., 2011; Morote et al., 2017), while others evidenced that negative emotions such as anxiety and depression had negative impacts on the development of resilience (Galatzer-Levy et al., 2013; Yu et al., 2015). In the challenge model of resilience (Garmezy et al., 1984; Fergus and Zimmerman, 2005), the influence of a risk factor on an outcome is curvilinear. This suggests that high levels and low levels of a risk factor are associated with negative outcomes, but moderate levels of the risk are related to less negative outcomes when adolescents can learn from the process of overcoming the risk. Similarly, according to the resiliency model (Richardson et al., 1990), an individual can be more resilient only if they learn from the negative experience with more coping skills. That is to say, without learning from the risks, there are still many risks and challenges which have a very negative influence on the development of resilience. For example, negative emotions could be viewed as destructive resources that hinder the development of resilience in adolescents. In other words, adolescents exposed to high levels of risk factors, such as negative emotions, might have low levels of resilience. On the basis of the challenge model of resilience and the resiliency model, it is reasonable to infer that higher levels of negative emotions, including depression, anxiety, and stress, result in lower levels of resilience, which further reduces adolescent mental well-being.

### Hypotheses of This Study

**FIGURE 1:**
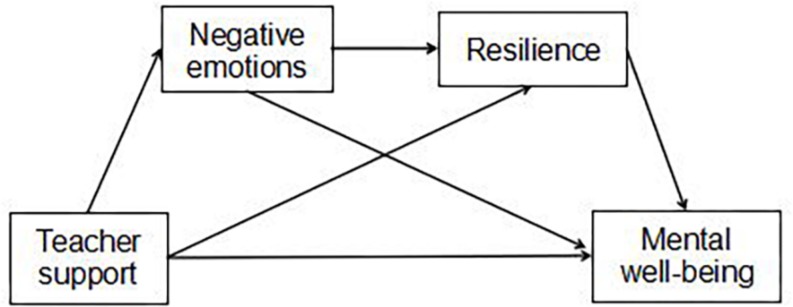
The proposed mediation model.

In this study, a serial mediation model (Figure 1) was proposed to test the mediating role of negative emotions and resilience in the association between teacher support and adolescent mental well-being. Specifically, four hypotheses (direct and indirect effects) were examined, as follows:

Hypothesis 1: Teacher support is directly associated with adolescent mental well-being.

Hypothesis 2: Teacher support is indirectly associated with adolescent mental well-being via negative emotions.

Hypothesis 3: Teacher support is indirectly associated with adolescent mental well-being via resilience.

Hypothesis 4: Teacher support is indirectly associated with adolescent mental well-being by negative emotions and then resilience.

## Materials and Methods

### Participants and Procedure

Participants were 1228 adolescents (652 male, 566 female, and 10 participants who gave no response as to their gender) enrolled at two junior middle schools and two senior middle schools in Tongling and Wuhu from Anhui Province in China. The four middle schools were selected by using convenience sampling. Two classes in each grade were randomly selected from each school. The final selection of 24 classes included 12 classes from junior middle school and 12 classes from senior middle school. Psychology teachers were contacted by the researchers, informed about the aim and procedure of the test, and asked to collaborate as experimenters, and were responsible for reading out the test introductions according to the standardized procedure. Adolescents completed a set of self-reported questionnaires in a class setting during the second semester of the 2017–2018 school year, which also included demographic information, including gender, age, and grade.

The final sample consisted of 1228 Chinese adolescents (611 junior school students and 617 senior school students) aged 11–20 years. The proportion of participants from grades 1 to 3 in junior middle school and senior middle school was 15.47, 16.78, and 17.51% and 16.86, 16.53, and 16.86%, respectively. The proportion of male and female was 53.09 and 46.09%, respectively. The mean age of participants was 15.43 years (*SD* = 1.76). All participants and their parents provided written informed consent before the study. The study protocol received approval from the Ethics Committee of Anhui Normal University.

### Instruments

#### Teacher Support

The Students’ Perception of Teacher’s Behavioral Support Questionnaire (SPTBSQ) is a 19-item instrument designed to assess teacher support (Ouyang, 2005). The SPTBSQ measures students’ perception of teacher’s behavioral support in their studies and life. Items are rated on a six-point scale ranging from 1 (*was not relevant to me at all*) to 6 (*was very relevant to me*), and students were asked to indicate to what extent each statement applied to them. The questionnaire contains three dimensions, including learning support (nine items, such as “when I can’t answer the question, my teacher often repeats the question to me”), emotional support (six items, such as “my teacher often encourages me in my study and life”), and ability support (four items, such as “my teacher often recommends that I participate in various activities or competitions”). Specifically, learning or academic support reflects students’ belief that teachers care about what and how much they have learned, emotional support describes students’ feelings that teachers care about their life and are kind to them, and ability support refers to students’ perception that teachers encourage them to participate different activities and contest (Ouyang, 2005; Vervoort et al., 2014; Ansong et al., 2017; Liu et al., 2018). Item 11 is a negative item. After reverse-coding this item, it is possible to obtain a global measure of teacher support by estimating the mean of 19 items, with higher scores indicating higher teacher support. The original SPTBSQ has been shown to have an adequate reliability (Cronbach’s alpha of 0.87) and three-factor structure (three factors accounted for 50.64% of the variation). The SPTBSQ has also been shown to have an adequate reliability when completed by middle school students (Cronbach’s alpha ranging from 0.82 to 0.89) and construct validity (Qiao et al., 2013; Chen and Guo, 2016; Chen et al., 2018). In the current sample, Cronbach’s alpha for the entire questionnaire was 0.90, and ranged from 0.71 to 0.84 for the emotional support, ability support, and learning support sub-scales.

#### Negative Emotions

Negative emotions were evaluated using the short-form version of the Depression Anxiety Stress Scale (Lovibond and Lovibond, 1995), translated into Chinese. The Chinese version of DASS-21 is an effective instrument to measure negative emotions (Gong et al., 2010; Wang et al., 2016). Participants are asked to respond to each item by rating the frequency and severity of the symptoms experienced over the past week using a 4-point Likert scale (0 = *did not apply to me at all*, 3 = *applied to me very much or most of the time*). The scale contains three dimensions, including depression (seven items, such as “I felt that I had nothing to look forward to”), anxiety (seven items, such as “I felt I was close to panic”), and stress (seven items, such as “I found it difficult to relax”). The total score is the mean of all items, with higher scores indicating higher negative emotions. The original DASS-21 (Antony et al., 1998) has been shown to have an adequate reliability (Cronbach’s alphas for the DASS-21 sub-scales were 0.94 for depression, 0.87 for anxiety, and 0.91 for stress) and excellent structures (three factors accounted for 67.00% of the variation). The Chinese version of the DASS-21 has also been reported to have an adequate reliability (Cronbach’s alpha, 0.89) and construct validity (Gong et al., 2010). In the current sample, Cronbach’s alpha for the entire questionnaire was 0.92, and ranged from 0.77 to 0.86 for the depression, stress, and anxiety sub-scales.

#### Resilience

The Resilience Scale for Chinese Adolescents (Hu and Gan, 2008) is a localized resilience scale specifically for Chinese adolescents. All items were developed on the basis of the resiliency model (Richardson et al., 1990) which contains individual experience and envirosocial influences. The scale items are rated on a five-point scale, ranging from 1 (*did not apply to me at all*) to 5 (*applied to me very much*), and students are asked to indicate how much the statement applies to them when they are faced with adversity and discouragement. The scale includes 27 items that are classified into five factors, including goal planning (five items, such as “I tend to be more mature and experienced after experiencing setbacks”), affect control (six items, such as “I have difficulty in controlling my unpleasant emotions”), positive thinking (four items, such as “I think adversity has an incentive effect on people”), family support (six items, such as “my parents respect my opinions very much”), and help-seeking behavior (six items, such as “I have a friend with whom I can talk about my difficulties”). After reverse-coding negative items (items 1, 2, 5, 6, 9, 12, 15, 16, 17, 21, 26, and 27), a global measure of resilience can be obtained by calculating the mean score of all 27 items, with higher scores indicating higher resilience. The original scale has been reported to have an adequate reliability (Cronbach’s alpha, 0.85) and five-factor structure (five factors accounted for 52.40% of the variation). In the current sample, Cronbach’s alpha for the entire questionnaire was 0.85, and ranged from 0.72 to 0.81 for the goal planning, affect control, positive thinking, family support, and help-seeking behavior subscales.

#### Mental Well-Being

Students’ mental well-being was assessed using the Warwick-Edinburgh Mental Well-Being Scale (WEMWBS; Tennant et al., 2007), translated into Chinese (Liu et al., 2016). The scale comprises 14 positively phrased items (e.g., “I’ve been feeling useful,” “I’ve been able to make up my own mind about things,” and “I’ve been feeling cheerful”), and measures the positive aspects of mental well-being in the previous 2 weeks. Each item is scored on a five-point scale ranging from 1 (*none of the time*) to 5 (*all of the time*), with higher scores indicating better levels of mental well-being. The original scale has been found to have an adequate reliability (Cronbach’s alphas for a student sample and population sample were 0.89 and 0.91, respectively), test–retest reliability (*r* = 0.83), and construct validity (confirmatory factor analysis supported the single factor hypothesis). The Chinese version of the scale (Liu et al., 2016) has also been reported to exhibit an adequate reliability (Cronbach’s alpha, 0.93) and test–retest reliability (*r* = 0.79). And a previous study showed that the Chinese version of WEMWBS had good reliability and validity for the assessment of mental well-being in Chinese adolescents (Zhao et al., 2019). In the current sample, Cronbach’s alpha for the entire questionnaire was 0.88.

### Statistical Analyses

The statistical analyses were conducted using the Statistical Package for the Social Sciences (SPSS, version 21.0). Descriptive statistics were computed for demographic data and all study variables. The associations between variables were assessed by Pearson’s bivariate correlation, and the strength of these associations was classified according to the following standard: “small” for correlations around 0.10, “medium’ for correlations near 0.30, and “large” for those at 0.50 or higher (Cohen, 1988). For testing mediating effects, the method of the bias-corrected bootstrap provides the most accurate confidence interval (CI) estimation and has the highest statistical efficacy (Fang et al., 2012). Therefore, in the current study, a bootstrapping analysis was conducted using the SPSS macro PROCESS Model 6 (with teacher support as the independent variable, mental well-being as the outcome variable, negative emotions and resilience as mediators, and gender and age as covariates) with 5000 resamples to test a serial mediation model, and to calculate the 95% CIs. Even more importantly, it is necessary to know to what extent do the three dimensions of negative emotions and the five factors of resilience contribute to the relationship between teacher support and mental well-being. Therefore, on the premise of the significant mediation of negative emotions and resilience, a bootstrapping analysis was conducted using the SPSS macro PROCESS Model 4 (with teacher support as the independent variable, mental well-being as the outcome variable, the three dimensions of negative emotions and the five factors of resilience as mediators, and gender and age as covariates) with 5000 resamples to test a parallel mediation. The indirect effect was considered statistically significant if the 95% bias-corrected CI did not contain zero (Hayes, 2013).

## Results

### Descriptive and Pearson’s Correlation Results

The descriptive statistics and Pearson’s correlations for all of the assessed variables are presented in Table 1. Specifically, mental well-being was positively and strongly associated with both teacher support (*r* = 0.47, *p* < 0.01) and resilience (*r* = 0.58, *p* < 0.01). Likewise, a positive and strong relationship was also observed between teacher support and resilience (*r* = 0.46, *p* < 0.01). In addition, negative emotions were negatively and moderately correlated with both mental well-being (*r* = −0.39, *p* < 0.01) and teacher support (*r* = −0.19, *p* < 0.01). Negative emotions were negatively and strongly correlated with resilience (*r* = −0.58, *p* < 0.01).

**TABLE 1:** Descriptive statistics and Pearson’s correlations between the study variables.

**Variables**	***M***	***SD***	**1**	**2**	**3**	**4**	**5**	**6**	**7**	**8**	**9**	**10**	**11**	**12**	**13**
1. Gender	0.54	0.50	–												
2. Age	15.43	1.76	–0.01	–											
3. Mental well-being	3.47	0.60	0.07^∗^	–0.12^∗∗^	–										
4. Teacher support	3.81	0.85	–0.06	–0.16^∗∗^	0.47^∗∗^	–									
5. Resilience	3.34	0.55	−0.07^∗^	–0.04	0.58^∗∗^	0.46^∗∗^	–								
6. Goal planning	3.43	0.74	0.03	–0.13^∗∗^	0.51^∗∗^	0.44^∗∗^	0.63^∗∗^	–							
7. Affect control	3.08	0.83	0.08^∗∗^	−0.07^∗^	0.46^∗∗^	0.22^∗∗^	0.68^∗∗^	0.28^∗∗^	–						
8. Positive thinking	3.93	0.77	–0.01	–0.03	0.39^∗∗^	0.33^∗∗^	0.58^∗∗^	0.54^∗∗^	0.28^∗∗^	–					
9. Family support	3.35	0.83	–0.15^∗∗^	0.14^∗∗^	0.26^∗∗^	0.30^∗∗^	0.65^∗∗^	0.26^∗∗^	0.27^∗∗^	0.22^∗∗^	–				
10. Help-seeking behavior	3.11	0.98	–0.14^∗∗^	–0.08^∗∗^	0.32^∗∗^	0.27^∗∗^	0.69^∗∗^	0.21^∗∗^	0.31^∗∗^	0.16^∗∗^	0.29^∗∗^	–			
11. Negative emotions	0.93	0.56	0.04	–0.05	–0.39^∗∗^	–0.19^∗∗^	–0.58^∗∗^	–0.29^∗∗^	–0.55^∗∗^	–0.29^∗∗^	–0.38^∗∗^	–0.33^∗∗^	–		
12. Depression	0.76	0.65	0.06^∗^	–0.03	–0.43^∗∗^	–0.21^∗∗^	–0.58^∗∗^	–0.35^∗∗^	–0.47^∗∗^	–0.34^∗∗^	–0.39^∗∗^	–0.34^∗∗^	0.89^∗∗^	–	
13. Anxiety	0.93	0.60	0.00	−0.06^∗^	–0.31^∗∗^	–0.14^∗∗^	–0.47^∗∗^	–0.21^∗∗^	–0.45^∗∗^	–0.22^∗∗^	–0.32^∗∗^	–0.28^∗∗^	0.91^∗∗^	0.70^∗∗^	–
14. Stress	1.11	0.61	0.03	–0.04	–0.32^∗∗^	–0.15^∗∗^	–0.51^∗∗^	–0.21^∗∗^	–0.57^∗∗^	–0.22^∗∗^	–0.31^∗∗^	–0.28^∗∗^	0.91^∗∗^	0.69^∗∗^	0.77^∗∗^

*Gender: male = 1, female = 0; goal planning, affect control, positive thinking, family support, and help-seeking behavior belong to resilience. Depression, anxiety, and stress belong to negative emotions.^∗^*p* < 0.05, ^∗∗^*p* < 0.01.*

### Testing for a Serial Mediation Model

**FIGURE 2:**
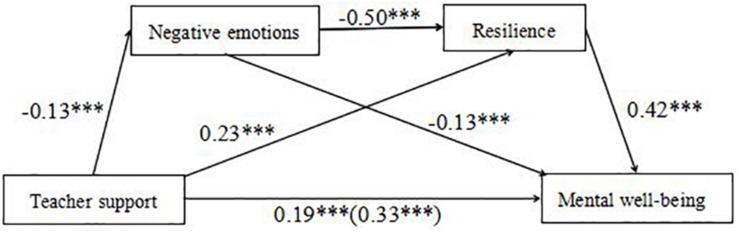
Serial mediation model shows effects of teacher support, negative emotions, and resilience on mental well-being. *N* = 1199. The total effect of teacher support is shown in parentheses. Regression coefficients were obtained after controlling for age and gender in PROCESS Procedure for SPSS. ^∗∗∗^*p* < 0.001.

We tested a serial mediation model, which consisted of three indirect effects, as follows: (1) teacher support enhances mental well-being via negative emotions, (2) teacher support enhances mental well-being via resilience, and (3) teacher support enhances mental well-being via negative emotions and then resilience (Figure 2).

After controlling for the effects of age and gender, the results showed a negative effect of teacher support on negative emotions, *B* = −0.13, *t* = −7.00, *p* < 0.001, and a positive effect of teacher support on resilience, *B* = 0.23, *t* = 16.76, *p* < 0.001. There was a negative relationship between negative emotions and resilience, *B* = −0.50, *t* = −23.67, *p* < 0.001. Moreover, both negative emotions and resilience significantly predicted mental well-being, *B* = −0.13, *t* = −4.56, *p* < 0.001 for negative emotions and *B* = 0.42, *t* = 12.63, *p* < 0.001 for resilience. The total effect of teacher support on mental well-being was statistically significant, *B* = 0.33, *t* = 18.37, *p* < 0.001. The direct effect of teacher support on mental well-being was also significant, even after controlling for the effects of negative emotions, resilience, age, and gender, *B* = 0.19, *t* = 10.43, *p* < 0.001.

Furthermore, the indirect effect of teacher support on mental well-being through negative emotions was significant, *B* = 0.018, SE = 0.006, 95% CI (0.008, 0.030). The mediation effect (teacher support → negative emotions → mental well-being) accounted for 5.45% of the total effect. Also, resilience mediated the relationship between teacher support and mental well-being, *B* = 0.099, SE = 0.010, 95% CI (0.080, 0.121). The mediation effect (teacher support → resilience → mental well-being) accounted for 30.00% of the total effect. Finally, the indirect effect of teacher support on mental well-being through negative emotions and then resilience (i.e., a serial mediating effect) was also found, *B* = 0.028, SE = 0.005, 95% CI (0.019, 0.038). The mediation effect (teacher support → negative emotions → resilience → mental well-being) accounted for 8.48% of the total effect. The direct and indirect effects of negative emotions and resilience on the relationship between teacher support and mental well-being are shown in Table 2.

**TABLE 2:** Direct, indirect, and total effects of teacher support on mental well-being.

**Model pathways**	**Estimated effect (β)**	**95% CI**	
		**Lower**	**Upper**
**Direct effect**			
TS → MWB	0.186^∗∗∗^	0.151	0.221
**Indirect effects**			
TS → NE → MWB	0.018^∗∗^	0.008	0.030
TS → RE → MWB	0.099^∗∗^	0.080	0.121
TS → NE → RE → MWB	0.028^∗∗^	0.019	0.038
Total effect	0.144^∗∗^	0.121	0.170

*TS, teacher support; NE, negative emotions; RE, resilience; MWB, mental well-being; ^∗∗^*p* < 0.01, ^∗∗∗^*p* < 0.001.*

Since the three indirect effects (including the mediation effect of negative emotions in the relationship between teacher support and mental well-being, the mediation effect of resilience in the relationship between teacher support and mental well-being, and the serial mediation effect of negative emotions and resilience in the relationship between teacher support and mental well-being) were statistically significant, we examined whether these effects were significantly different in the mediation effects. There was no significant difference between the mediating effect of negative emotions and the serial mediating effect of negative emotions and then resilience, *B* = −0.010, SE = 0.007, 95% CI (−0.025, 0.002). However, the mediating effect of negative emotions was weaker than the mediating effect of resilience, *B* = −0.081, SE = 0.014, 95% CI (−0.109, −0.054). Similarly, the mediating effect of resilience was stronger than the serial mediating effect of negative emotions and then resilience, *B* = −0.071, SE = 0.010, 95% CI (−0.092, −0.053).

### Testing for a Parallel Mediation Model

**FIGURE 3:**
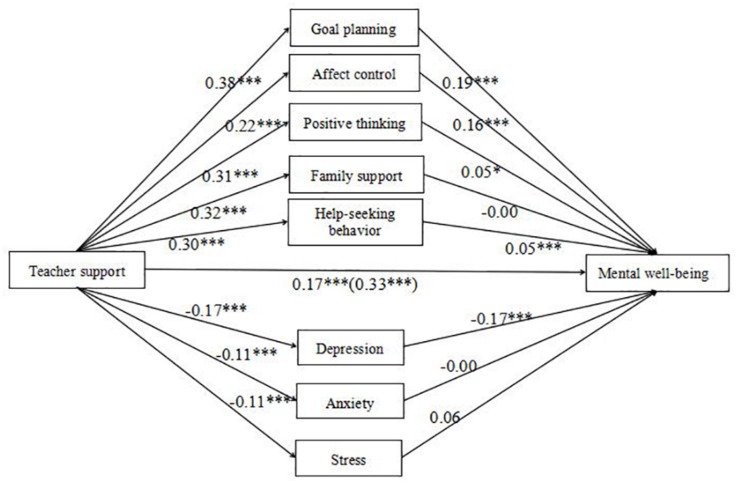
Parallel mediation model showing the individual dimensions of negative emotions and resilience in the relationship between teacher support and mental well-being. *N* = 1199. The total effect of teacher support is shown in parentheses. Goal planning, affect control, positive thinking, family support, and help-seeking behavior belong to resilience. Depression, anxiety, and stress belong to negative emotions. ^∗^*p* < 0.05, ^∗∗∗^*p* < 0.001.

After controlling for the effects of age and gender, the results of parallel mediation are shown in Figure 3. Specially, teacher support had a positive effect on goal planning, *B* = 0.38, *t* = 16.78, *p* < 0.001; affect control, *B* = 0.22, *t* = 7.86, *p* < 0.001; positive thinking, *B* = 0.31, *t* = 12.48, *p* < 0.001; family support, *B* = 0.32, *t* = 12.08, *p* < 0.001; and help-seeking behavior, *B* = 0.30, *t* = 9.16, *p* < 0.001; and a negative effect on depression, *B* = −0.17, *t* = −7.77, *p* < 0.001; anxiety, *B* = −0.11, *t* = −5.64, *p* < 0.001; and stress, *B* = −0.11, *t* = −5.37, *p* < 0.001. Mental well-being was significantly predicted by goal planning, *B* = 0.19, *t* = 8.22, *p* < 0.001; affect control, *B* = 0.16, *t* = 8.08, *p* < 0.001; positive thinking, *B* = 0.05, *t* = 2.25, *p* < 0.05; help-seeking behavior, *B* = 0.05, *t* = 3.54, *p* < 0.001; and depression, *B* = −0.17, *t* = −5.23, *p* < 0.001. The direct effect of teacher support on mental well-being was significant, *B* = 0.17, *t* = 9.79, *p* < 0.001.

Furthermore, the indirect effect of teacher support on mental well-being through goal planning was significant, *B* = 0.071, SE = 0.011, 95% CI (0.051, 0.094). The mediation effect (teacher support → goal planning → mental well-being) accounted for 21.52% of the total effect. Also, affect control mediated the relationship between teacher support and mental well-being, *B* = 0.035, SE = 0.007, 95% CI (0.023, 0.050). The mediation effect (teacher support → affect control → mental well-being) accounted for 10.61% of the total effect. In addition, help-seeking behavior mediated the relationship between teacher support and mental well-being, *B* = 0.015, SE = 0.005, 95% CI (0.007, 0.025). The mediation effect (teacher support → help-seeking behavior → mental well-being) accounted for 4.55% of the total effect. However, the indirect effect of teacher support on mental well-being through positive thinking was not significant, *B* = 0.014, SE = 0.008, 95% CI (−0.000, 0.029). The indirect effect of teacher support on mental well-being through family support was not significant, *B* = −0.001, SE = 0.006, 95% CI (−0.013, 0.011). Finally, the indirect effect of teacher support on mental well-being through depression was also found, *B* = 0.028, SE = 0.008, 95% CI (0.014, 0.046). The mediation effect (teacher support → depression → mental well-being) accounted for 8.48% of the total effect. The indirect effect of teacher support on mental well-being through anxiety was not significant, *B* = 0.000, SE = 0.004, 95% CI (−0.008, 0.010). Also, the indirect effect of teacher support on mental well-being through stress was not significant, *B* = −0.006, SE = 0.005, 95% CI (−0.016, 0.001). The direct and indirect effects of the three dimensions of negative emotions and the five factors of resilience on the relationship between teacher support and mental well-being are shown in Table 3.

**TABLE 3:** Direct, indirect, and total effects of teacher support on mental well-being in the parallel mediation.

**Model pathways**	**Estimated effect (β)**	**95% CI**	
		**Lower**	**Upper**
**Direct effect**			
TS → MWB	0.174^∗∗∗^	0.139	0.208
**Indirect effects**			
TS → goal planning → MWB	0.071^∗∗^	0.051	0.094
TS → affect control → MWB	0.035^∗∗^	0.023	0.050
TS → positive thinking → MWB	0.014	–0.000	0.029
TS → family support → MWB	–0.001	–0.013	0.011
TS → help-seeking behavior → MWB	0.015^∗∗^	0.007	0.025
TS → depression → MWB	0.028^∗∗^	0.014	0.046
TS → anxiety → MWB	0.000	–0.008	0.010
TS → stress → MWB	–0.006	–0.016	0.001
Total effect	0.156^∗∗^	0.131	0.185

*TS, teacher support; RE, resilience; MWB, mental well-being; goal planning, affect control, positive thinking, family support, and help-seeking behavior belong to resilience. Depression, anxiety, and stress belong to negative emotions. ^∗∗^*p* < 0.01, ^∗∗∗^*p* < 0.001.*

Since the four indirect effects (including the mediation effect of goal planning, affect control, help-seeking behavior, and depression in the relationship between teacher support and mental well-being) were statistically significant, we examined whether these effects were significantly different in the mediation effects. There was no significant difference between the mediating effect of depression and the mediating effect of affect control, *B* = −0.007, SE = 0.010, 95% CI (−0.027, 0.013). Similarly, there was no significant difference between the mediating effect of depression and the mediating effect of help-seeking behavior, *B* = 0.013, SE = 0.010, 95% CI (−0.005, 0.033). However, the mediating effect of depression was weaker than the mediating effect of goal planning, *B* = −0.042, SE = 0.014, 95% CI (−0.071, −0.016). The mediating effect of goal planning was stronger than the mediating effect of affect control, *B* = 0.036, SE = 0.012, 95% CI (0.012, 0.061), and the mediating effect of help-seeking behavior, *B* = 0.055, SE = 0.012, 95% CI (0.032, 0.082). In addition, the mediating effect of affect control was stronger than the mediating effect of help-seeking behavior, *B* = 0.020, SE = 0.009, 95% CI (0.004, 0.037).

## Discussion

The results of this study show that the mediating effects of negative emotions and resilience may contribute to understanding the relationship between teacher support and mental well-being in a sample of Chinese adolescents.

First, consistent with a prior study (Guo et al., 2018), we found that teacher support was a significant predictor of mental well-being. Together, this indicates that adolescents who receive more care and help from teachers tend to have a high level of mental well-being. This also provides further evidence for the positive effect of teacher support on adolescent mental well-being. Moreover, our results support our second hypothesis that negative emotions represent a potential underlying mechanism that could partially explain how teacher support is linked with adolescent mental well-being. The associations between teacher support, negative emotions, and mental well-being can be explained by the buffering model of social support (Gong, 1994). Teacher support, as a kind of social support, can keep adolescents healthy by reducing the influence of negative emotions on the body and mind. For example, when adolescents get along well with teachers and receive more care and help from teachers, they are more likely to feel that they are in a safe and friendly environment, which decreases negative emotions such as depression (Joyce and Early, 2014). Adolescents who receive more help from teachers also tend to have more self-awareness and positive self-evaluation. As a result, they may experience fewer negative emotions, especially lower depression. For example, researchers found that teacher support was negatively associated with depression in adolescents (Mizuta et al., 2017; Pössel et al., 2018). On the other hand, Moksnes et al. (2014) reported that negative emotions (e.g., depression) had a significant and negative association with mental well-being (e.g., life satisfaction) in adolescents. In the same way, adolescents who have a negative self-perception accompanied by depression, anxiety, and stress are more likely to have a low subjective evaluation of life satisfaction, which could be a predictor of mental well-being (Freire and Ferreira, 2016). Together, these observations suggest that adolescents who receive more help from teachers will tend to experience less negative emotions and be more satisfied with life, resulting in a higher level of mental well-being.

Second, in accordance with the framework of resilience in action (Li and Zhang, 2006) and the self-determination theory (Ryan and Deci, 2000), we identified a mediating role of resilience on the association between teacher support and adolescent mental well-being. In other words, promoting teacher support as a way to build up resilience could help to improve adolescent mental well-being. According to previous studies (Liebenberg et al., 2016; Rosenberg et al., 2018), teachers can encourage adolescents to build resilience by establishing good quality teacher–student interactions and relationships. Adolescents with a high level of resilience are more likely to have confidence in dealing with adversity and challenges, and to be able to cope with difficulties; they tend to evaluate the self-perception of mental well-being with a positive attitude. Ho et al. (2015) also found that resilience can improve mental health in Hong Kong Chinese adolescents.

Third, in terms of our findings with the parallel mediation, teacher support improves adolescent mental well-being by increasing goal planning, affect control, and help-seeking behavior, and decreasing depression. That is, teachers can promote adolescents mental well-being by teaching them how to learn from individual experience, control themselves, ask for help, and regulate their negative emotions, especially depression. A 5-year longitudinal study evidenced that the decreased depression was predicted by increasing teacher support 1 year before (Pössel et al., 2013). In a word, teachers play a crucial role in adolescent healthy development. On the one hand, teachers provide adolescents with support that can be beneficial for improving adolescent mental well-being. On the other hand, teachers can also make a great contribution to increasing adolescent goal planning, affect control, and help-seeking behavior, and decreasing depression. What’s more, in order to improve a higher level of adolescent mental well-being, teachers and schools ought to pay due attention to the specific aspects of resilience (i.e., goal planning, affect control, and help-seeking behavior) and negative emotions (i.e., depression). With more attention and help from teachers, adolescents can possess a higher level of mental well-being.

Finally, for the first time, we found support for the serial mediation model. Teacher support was indirectly associated with adolescent mental well-being through negative emotions and then resilience. Similarly, prior studies have consistently demonstrated that the current stressor and lower negative emotions can negatively influence resilience, and subsequently reduce the protective effect of resilience on mental health (Galatzer-Levy et al., 2013; Kim et al., 2015). In other words, adolescents who experience strong negative emotions may abandon themselves to sadness and feelings of misery, which undermine initiative and enthusiasm to solve problems. Generally speaking, this means that they cannot actively adapt to frustrations and difficulties and the building up of higher resilience levels is therefore inhibited. Furthermore, based on the framework of resilience in action (Li and Zhang, 2006), adolescents who experience less negative emotions are more likely to be able to generate happy feelings and experience satisfaction with life, which in turn will assist them in regulating their behavior and especially their emotions. Once adolescents become capable of emotional regulation, they can develop more confidence and inner resources, such as problem solving, self-consciousness, and self-efficacy, which can promote the development of resilience (Liu and Ngai, 2018).

The present study has several limitations that should be addressed. First, cross-sectional designs do not allow us to make causal conclusions about the relationships between variables, although our findings support the possibility of such a relationship. Experimental or longitudinal studies could be carried out in the future to further investigate the causality of the relationships identified in the present work. Second, our results cannot be generalized to students in other grades or adolescents from other cultural backgrounds. Thus, future studies could use more diverse samples, such as those of different ages and from different cultures. Third, the present research focused on teacher support and did not consider the influence of social support from friends. In fact, peer acceptance could weaken the relationship between negative self-cognition and adolescent depression (Tang et al., 2018). Future work should therefore investigate the influence of peer support on mental well-being.

Despite these shortcomings, this study contributes to existing research into the relationship between teacher support and adolescent mental well-being, at least in China, and sheds light on similar topics in other cultures. Additionally, our findings provide insights about the steps that could be taken to improve adolescent mental well-being. As far as teacher–student relationships are concerned, schools could develop a prevention program to inform teachers that their care and help are extremely important for adolescent mental well-being. Hence, teachers should aim to build good teacher–student relationships and help adolescents with patience and kindness. In terms of the mediating role of negative emotions, schools should place an importance on good interactions and communication between teachers and adolescents, and encourage teachers to provide learning or emotional support that can help adolescents experience less negative emotions. Both schools and teachers should focus on adolescents’ emotional changes and teach them different ways to regulate their negative emotions, especially depression. Researchers pointed that the process of affect (such as anxiety, joy, depression, and so on) regulation is applicable to promotion and restoration of mental well-being (Quoidbach et al., 2015; Gross et al., 2019). Moreover, our findings support the view that resilience plays an important role in the association between negative emotions and mental well-being. Thus, schools should also promote the development of adolescent resilience through activities and training, such as emotional regulation programs, courses related with goal planning and help-seeking behavior, which would help to further improve the subjective perception of mental well-being.

## Data Availability Statement

The raw data supporting the conclusions of this article will be made available by the authors, without undue reservation, to any qualified researcher.

## Ethics Statement

The studies involving human participants were reviewed and approved by the Anhui Normal University. Written informed consent to participate in this study was provided by the participants’ legal guardian/next of kin.

## Author Contributions

JG: guarantor of integrity of entire study, study concepts, study design, literature research, data acquisition, data analysis/interpretation, statistical analysis, manuscript preparation, manuscript revision, and manuscript final version approval. LL: literature research, manuscript editing, manuscript revision, and manuscript final version approval. BZ: literature research, data interpretation, statistical analysis, manuscript preparation, and manuscript final version approval. DW: literature research, guarantor of integrity of the entire study, manuscript definition of intellectual content, manuscript editing, manuscript revision, and manuscript final version approval.

## Conflict of Interest

The authors declare that the research was conducted in the absence of any commercial or financial relationships that could be construed as a potential conflict of interest.
